# Genetic connectivity among swarming sites in the wide ranging and recently declining little brown bat (*Myotis lucifugus*)

**DOI:** 10.1002/ece3.1266

**Published:** 2014-10-12

**Authors:** Lynne E Burns, Timothy R Frasier, Hugh G Broders

**Affiliations:** 1 Department of Biology, Dalhousie University, Life Sciences Centre1355 Oxford Street, Halifax, Nova Scotia, B3H 4J1, Canada; 2 Department of Biology, Saint Mary's University923 Robie Street, Halifax, Nova Scotia, B3H 3C3, Canada

**Keywords:** Chiroptera, dispersal, genetic structure, mating, *Myotis*, swarming

## Abstract

Characterizing movement dynamics and spatial aspects of gene flow within a species permits inference on population structuring. As patterns of structuring are products of historical and current demographics and gene flow, assessment of structure through time can yield an understanding of evolutionary dynamics acting on populations that are necessary to inform management. Recent dramatic population declines in hibernating bats in eastern North America from white-nose syndrome have prompted the need for information on movement dynamics for multiple bat species. We characterized population genetic structure of the little brown bat, *Myotis lucifugus*, at swarming sites in southeastern Canada using 9 nuclear microsatellites and a 292-bp region of the mitochondrial genome. Analyses of *F*_ST_, Φ_ST,_ and Bayesian clustering (STRUCTURE) found weak levels of genetic structure among swarming sites for the nuclear and mitochondrial genome (Global *F*_ST_ = 0.001, *P* < 0.05, Global Φ_ST_ = 0.045, *P* < 0.01, STRUCTURE *K* = 1) suggesting high contemporary gene flow. Hierarchical AMOVA also suggests little structuring at a regional (provincial) level. Metrics of nuclear genetic structure were not found to differ between males and females suggesting weak asymmetries in gene flow between the sexes. However, a greater degree of mitochondrial structuring does support male-biased dispersal long term. Demographic analyses were consistent with past population growth and suggest a population expansion occurred from approximately 1250 to 12,500 BP, following Pleistocene deglaciation in the region. Our study suggests high gene flow and thus a high degree of connectivity among bats that visit swarming sites whereby mainland areas of the region may be best considered as one large gene pool for management and conservation.

## Introduction

Understanding the structure and dynamics of populations has long been recognized as a foundation for informing management decisions for species at risk because it provides the essential evolutionary perspective to the conservation process (Frankel 1974). Population genetics can be used in conservation efforts in delineating management units, management of captive or populations in decline, population reintroductions, and lastly in understanding past population demographics (Frankham et al. 2002; Pearse and Crandall 2004). Advances in population genetic theory and statistical analyses allow for inferences on historical demography to be made yielding greater insight into the processes that have led to contemporary patterns of genetic variability and population structuring (Avise et al. 1988; Rogers and Harpending 1992). To incorporate these insights into management strategies, a critical first step is characterizing the patterns of genetic variation. From there, inference can be made on gene flow and extent of genetic connectivity within and among populations (Slatkin 1994; Lowe and Allendorf 2010). Characterizing population structure remains an important step for conservation planning for many wildlife populations where detailed demographic data are limited and conservation risks are high.

The degree of connectivity within and among populations is influenced by environmental and biotic factors and traits specific to species such as dispersal. Dispersal is the movement of individuals from their natal group to a breeding group such that genetic exchange has occurred (Allendorf and Luikart 2007). Dispersal has been quantified through field studies of individuals using observation methods such as mark–recapture studies or telemetry to infer movements (e.g., Lebreton et al. 2003; Russell et al. 2005b; Hassall and Thompson 2012; Hoogland 2013; Schofield et al. 2013). However, identifying individual dispersers or the actual movements that lead to gene flow is difficult, especially for species that are highly vagile, cryptic, or long-lived. Assessment of dispersal and population genetic connectivity via molecular techniques can overcome these challenges by providing evidence of genetic exchange. This has been demonstrated for many vagile vertebrate taxa (e.g., Lyrholm et al. 1999; Petit and Mayer 1999; Wright et al. 2005).

As the only mammalian order capable of true powered flight, bats (Order Chiroptera) have high vagility (Fenton 1997) with many species engaging in long-distance movements during seasonal migrations ranging from tens to over a thousand kilometers (Fleming and Eby 2003; Hutterer et al. 2005). High vagility has facilitated large distributional ranges for many species. For several species, individuals may disperse over long distances resulting in high rates of gene flow and near panmictic population structuring (McCracken et al. 1994; Petit and Mayer 1999; Bryja et al. 2009). However, interspecific variation in the degree of philopatry, social structures, resource specializations, and mating systems causes variation in population structures (e.g., Burland et al. 1999; Kerth et al. 2002; Miller-Butterworth et al. 2003; Campbell et al. 2006; Rossiter et al. 2012). Because assessments of population genetic structure permit inference on population connectivity, particularly when combined with other demographic data (Lowe and Allendorf 2010), they can represent an important conservation tool for bats. This is particularly important for species considered at risk where data on movements, population dynamics, and connectivity are difficult to obtain efficiently to address urgent conservation concerns. Newly emergent threats to bat populations include high mortality as they migrate through wind farms (Cryan and Barclay 2009; Voigt et al. 2012; Hayes 2013), rapidly spreading novel diseases such as white-nose syndrome in North America (Blehert et al. 2009; Frick et al. 2010a). Data on movements and population connectivity are needed to understand and predict population-level impacts from such threats (Foley et al. 2011).

Temperate dwelling bats exhibit an annual cycle consisting of a lengthy period of reduced activity during hibernation, followed by a shorter active period used for self-maintenance and reproduction. For most of the active season, the sexes are segregated with females forming maternity colonies and males apparently living independently or in small groups (Safi 2008), although exceptions to complete segregation are known to exist (Altringham and Senior 2005). However, in the late summer and autumn, many species form mixed-sex aggregations composed of individuals from several colonies (Parsons and Jones 2003; Rivers et al. 2005; Furmankiewicz and Altringham 2007; Norquay et al. 2013) within which they engage in swarming activities. Swarming is the term used to describe the event of mass visitations by bats to underground sites prior to or just following hibernation. During swarming, bats engage in chasing and mating behaviors and presumably gather or exchange information that may include the suitability of hibernation sites or knowledge of migration routes and may include the orientation of young-of-the-year (YOY) to such sites (Davis 1964; Fenton 1969; Parsons et al. 2003; Piksa et al. 2011; Bogdanowicz et al. 2012a). Accumulating evidence such as copulations (Fenton 1969; Thomas et al. 1979), male-biased sex ratios, and observations of males in sexual condition (Gustafson and Damassa 1985; Entwistle et al. 1998; Kerth et al. 2003; Parsons et al. 2003), combined with recent genetic evidence (Veith et al. 2004; Rivers et al. 2005; Furmankiewicz and Altringham 2007; Bogdanowicz et al. 2012b), suggests that swarming is likely the primary mating period for many species. Mating can also occur at summer sites late in the season (e.g., Senior et al. 2005; Angell et al. 2013), en route to swarming sites, or during hibernation (Thomas et al. 1979). However, if significant mating occurs during swarming, it may play an important role in maintaining gene flow among individuals segregated during the summer by providing a mechanism for genetic exchange to occur among partially discrete summer bat populations.

**Figure 1:**
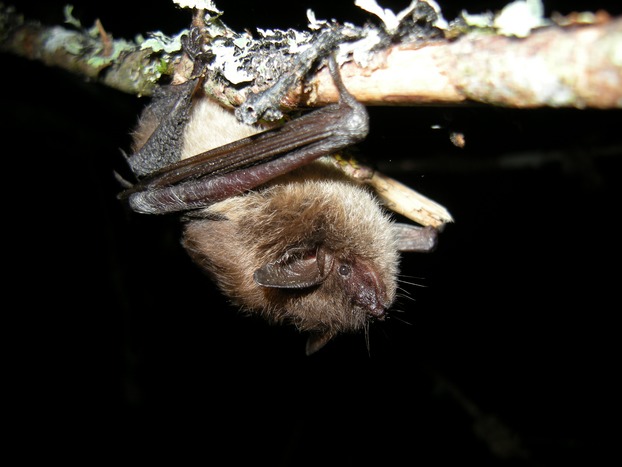
A male little brown bat (*Myotis lucifugus*) captured at swarming site in Nova Scotia, Canada. Photograph by Lynne Burns.

The little brown bat (*Myotis lucifugus*) is a small (6–10 g) temperate swarming species, distributed widely across North America (Fenton 1969; Schowalter 1980; Fig. 1). It is considered a roosting and dietary generalist species that roosts in buildings and trees in the summer and hibernates in caves and abandoned mines in the winter (Fenton and Barclay 1980; van Zyll de Jong 1985; Naughton 2012). Females form maternity colonies in the summer, and evidence suggests a high degree of fidelity to summer sites. However, complete philopatry does not occur as some females switch colonies (Davis and Hitchcock 1965; Humphrey and Cope 1976; Frick et al. 2010b; Norquay et al. 2013). This view is supported by recent genetic work that found low but significant population structure among maternity colonies in Minnesota suggesting some limited movements by individuals among the colonies (Dixon 2011). During the autumn, individuals make regional seasonal migration movements (hundreds of kilometers) between summer and winter/autumn sites where movements among swarming sites can occur within the same season and between years (Fenton 1969; Humphrey and Cope 1976). Norquay et al.'s (2013) banding data analysis found that *M. lucifugus* captured during swarming had the highest movement rates of all individuals studied (summer, winter, or swarming captured) which supports the contention that autumn swarming facilitates gene flow. Further, recent European studies showed higher genetic diversity at swarming sites compared to summering sites which supports the extra-colony hypothesis where multiple summering colonies fuse at swarming sites such that they act as mating centers (Kerth et al. 2003; Veith et al. 2004; Rivers et al. 2005). Taken together, these studies suggest that although there may be high gene flow in swarming species, different degrees of genetic structure may occur for different species depending on the specific vagility of species (e.g., distance of migratory movements) and landscape context. Structuring at swarming sites has not been previously investigated in any North American species including *M. lucifugus*, a known regional migrating species.

*Myotis lucifugus* is one of at least six species known to be susceptible to white-nose syndrome in North America, a newly emergent fungal disease of hibernating bats caused by the invasive species *Pseudogymnoascus destructans* (Blehert et al. 2009; Foley et al. 2011; Warnecke et al. 2012). Although the fungus is present in Europe, bats do not appear to suffer mass mortality from the disease (Puechmaille et al. 2011). *Myotis lucifugus* appears to have suffered significant mortality from the disease with severe population collapses reported within the affected region ranging from declines of 78 to 100% (Dzal et al. 2011; Langwig et al. 2012) where regional population extirpation is predicted within 20 years (Frick et al. 2010a). The degree to which the movements made during swarming contribute to the spread of the disease has not been quantified. However, owing to the rapid and unprecedented population decline of the species in the affected areas, information on the spatial extent of connectivity during this dynamic time period is needed. In addition to the summer colony study in Minnesota (Dixon 2011), previous published population genetic studies of *M. lucifugus* include a study in the western portion of the range using nuclear and mitochondrial markers that found little differentiation among summering areas suggesting high gene flow among putative subspecies in the sampled region (Lausen et al. 2008). Assessments of genetic variation among hibernacula have also found weak genetic differentiation suggesting high gene flow (Carmody et al. 1971; Miller-Butterworth et al. 2014).

Our goal was to determine to what degree swarming sites, or groups of sites, represent distinctive genetic clusters that could provide information relevant to establishing management priorities for the species. We quantified genetic variation in *M. lucifugus* among swarming sites in southeastern Canada using both mitochondrial DNA and nuclear microsatellite markers to investigate population genetic structure. Under the extra-colony mating hypothesis, swarming sites encompass individuals from a catchment area of summering bats where maternity colonies show site fidelity to swarming sites (e.g., Veith et al. 2004; Rivers et al. 2005). Therefore, we predicted to find on maternally inherited markers, higher genetic variation within swarming sites compared to among sites, and some level of structuring among sites if bats show site fidelity from summering areas to swarming sites. We further examined structuring on maternally inherited markers in the context of past demographic processes acting on the population to better understand any patterns observed. Previous tagging studies suggest movements among swarming sites can occur by some individuals during the autumn swarming season. As *M. lucifugus* can make extensive migration movements during the autumn mating season, we hypothesized there is a high degree of genetic connectivity among swarming sites even if the extra-colony hypothesis is occurring with some degree of swarming site fidelity by bats. We therefore predicted that genetic differentiation among swarming sites on nuclear markers would be lower than found on maternally inherited markers, suggesting gene flow occurs among sites regularly enough that we would find genetic clustering of multiple swarming sites rather than of individual swarming sites. Lastly, as previous work has found structuring on maternally inherited markers at hibernation sites (Miller-Butterworth et al. 2014), which is suggestive of restrictions on female movements and resultant gene flow, we tested for differences in asymmetries in gene flow among swarming sites where we expected to find a male bias in gene flow.

## Materials and Methods

### Sample collection and DNA extraction

**Figure 2:**
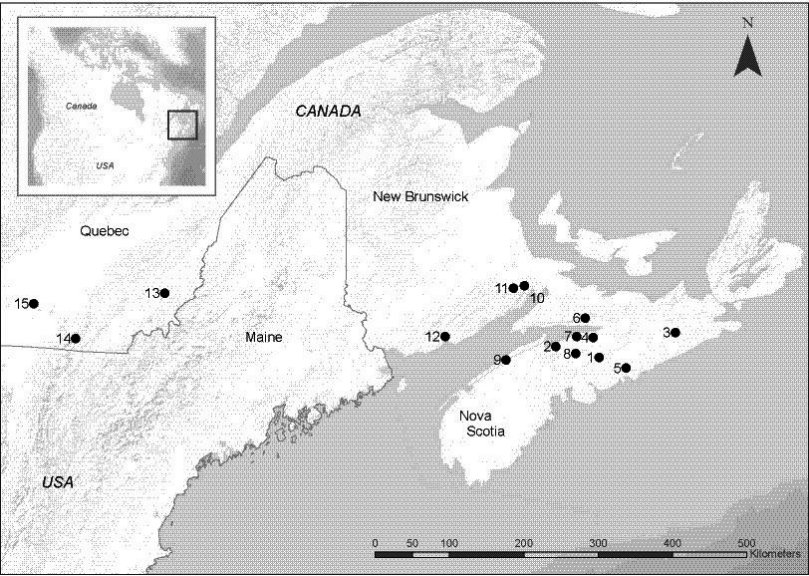
Sampling locations for *Myotis lucifugus* captured at swarming sites in southeastern Canada to assess population genetic structure. Geographic coordinates and names are not used due to the sensitive nature of swarming and hibernation sites; numbers correspond to site numbers in tables.

During the autumns of 2009–2011 (10 August to 06 October), bats were trapped in harp traps (Austbat Research Equipment, Lower Plenty, Victoria, Australia) or mist nets (Avinet, Dryden, New York) set at 15 swarming sites in three Canadian provinces: Quebec (QC), New Brunswick (NB), and Nova Scotia (NS; Table 1; Fig. 2). Sites were situated from 15 to 860 km from each other. Sites in NS and NB were selected as they were known swarming/hibernation sites. We included samples from QC to assess whether NS and NB were effectively one breeding group given the close proximity of sites to each other in these provinces. Precautionary WNS decontamination protocols provided by the US Fish and Wildlife Service were followed for all sampling using the most current protocol for each sampling season (available from http://whitenosesyndrome.org/topics/decontamination). Methods for the capture and handling of bats were approved by the Saint Mary's Animal Care Committee under permit from each provincial jurisdiction. White-nose syndrome was detected in winter 2009/2010 in southern counties of Quebec close to the sampling sites, and no further sampling was conducted. Detection of WNS in the winter of 2010/2011 in New Brunswick at one of the sites restricted our sampling to one site that was in a different county and did not have WNS detected at the time of sampling. We also reduced our trapping efforts in Nova Scotia in autumn 2011 to only sample sites where sample sizes were exceptionally low to reduce the risk of spreading the disease via the capture and handling of bats as WNS was not detected at the sites that autumn.

**Table 1:** Sampling site locations and numbers of individual *Myotis lucifugus* included in mitochondrial and nuclear microsatellites analyses

Site	Number of adults					Young-of-the-year	
	
	Province	mtDNA		Microsatellites		2009	2010
1	NS	18	(3 F/ 15 M)	25	(5 F/ 20 M)	5	7
2	NS	22	(11 F/ 11 M)	60	(25 F/ 35 M)	3	15
3	NS	29	(14 F/ 15 M)	70	(22 F / 48 M)	–	2
4	NS	23	(15 F/ 8 M)	126	(70 F / 56 M)	27	9
5	NS	30	(16 F/ 14 M	47	(24 F/ 23 M)	4	7
6	NS	30	(14 F/ 16 M)	54	(15 F/ 39 M)	8	8
7	NS	14	(5 F/ 9 M)	15	(5 F/ 10 M)	–	9
8	NS	31	(15 F/ 16 M)	88	(26 F/ 62 M)	17	23
9	NS	29	(14 F/ 15 M)	70	(23 F/ 47 M)	–	3
10	NB	27	(6 F/ 21 M)	29	(6 F / 23 M)	–	3
11	NB	28	(15 F/ 13 M)	40	(16 F/ 24 M)	–	15
12	NB	6	(3 F/ 3 M)	11	(4 F/ 7 M)	–	–
13	QC	28	(13 F/ 15 M)	60	(24 F/ 36 M)	–	–
14	QC	27	(10 F/ 17 M)	26	(7 F/ 19 M)	3	–
15	QC	14	(6 F/ 8 M)	14	(6 F/ 8 M)	–	–

Young-of-the-year was nuclear microsatellites only. NS, Nova Scotia; NB, New Brunswick; QC, Quebec.

For all captures, sex was identified, and age was determined as young-of-the-year (YOY) or adult based on the degree of ossification and fusion in the epiphyseal growth plates of the fourth metacarpal (Anthony 1988). Two small tissue samples (≈9 mm^2^ each) were collected from each of the wings of individuals (plagio or uropatagium; Faure et al. 2009; Broders et al. 2013) and then bats were released. Tissue samples were placed in either Allprotect Tissue Reagent (Qiagen Inc., Valencia, CA, USA) or 20% salt-saturated DMSO solution with 0.25 mol/L EDTA (Seutin et al. 1991), and stored frozen at −20°C. Tissues collected in Quebec were placed in 95% ethanol and stored at −20°C. In total, tissue samples were collected from 768 adults and 174 YOY. High-molecular-weight genomic DNA was extracted following a standard proteinase-K, phenol, and chloroform procedure followed by ethanol precipitation (Sambrook and Russell 2001). Extracted DNA was resuspended and diluted to approximately 5 ng/*μ*L in TE_0.1_ buffer (10 mmol/L Tris-Hcl (pH 8), 0.1 mmol/L EDTA, pH 8).

### Mitochondrial DNA sequencing

An approximate 300-base-pair (bp) fragment of the mitochondrial control region, hypervariable II domain (HV II), was amplified in 356 individuals (Table 1) using the previously described primer L16517 (Fumagalli et al. 1996) and primer KAHVII 5′-GTAGCGTGAATATGTCCTG-3′ (developed in-lab) which is internal to primer sH651 of Castella et al. (2001). Amplifications were carried out in 20 *μ*L reaction volumes containing 1× PCR Buffer (20 mmol/L Tris-HCl pH 8.4, 50 mmol/L KCl; Invitrogen, Carlsbad, CA, USA), 0.2 mmol/L of each dNTP (Invitrogen), 1.5 mmol/L MgCl2, 0.16 mg/mL Bovine Serum Albumin (Sigma Aldrich, St. Louis, MO, USA), 0.05 U/*μ*L *Taq* DNA polymerase, and approximately 10 ng of template DNA. The PCR amplification conditions were as follows: an initial denaturing cycle of 95°C for 5 min; followed by 30 cycles of 95°C for 30 sec, an annealing temperature of 55°C for 1 min, 72°C for 1 min; with a final extension period of 64°C for 45 min. The PCR products were purified using the Antarctic Phosphatase/Exonuclease I protocol (New England Biolabs, Ipswich, MA, USA). Sanger sequencing was performed with primer KAHVII using the Macrogen INC., Seoul, Korea Sequencing Service. All base calls were verified manually through visual examination of electropherograms, and sequences were trimmed to a common 292-bp segment using 4Peaks (v1.7) DNA sequence editing software (Griekspoor and Groothuis 2006).

### Tests of assumptions and genetic structuring on mtDNA

Mitochondrial DNA (mtDNA) sequences were aligned using Clustal W (Thompson et al. 1994) in the software MEGA (Tamura et al. 2011) using the default parameters following confirmation of congruence among alignments produced by doubling and halving the parameter settings. Sequences were then collapsed into haplotypes and formatted for downstream analysis using FaBox (Villesen 2007). To assess levels of genetic variation in the HV II domain, haplotype diversity (*h*; Nei 1987) and nucleotide diversity (*π*; Tajima 1983; Nei 1987) were calculated within each swarming site, on the whole dataset and for each sex separately using Arlequin v3.5.1.2 (Excoffier 2010).

To examine genetic differentiation among all swarming sites, Φ_ST_ values were calculated using Arlequin. Partitioning of genetic variation at a regional level for comparisons (i.e., among provinces) was examined using a hierarchical analysis of molecular variance (AMOVA) on adult females, adult males, and all adults. To determine what substitution model was most appropriate for our mitochondrial data, we ran ModelGenerator (Keane et al. 2006) on the mtDNA sequence data using the BIC and AIC selection criterion. This approach identified a version of the Kimura 2-parameter model (K80 + G; Kimura 1980) with a gamma distribution shape parameter estimate (*α*) of 0.11 and estimated transition/transversion rate ratio of 11.91. The genealogical relationships between mtDNA haplotypes were explored using a median-joining network (Bandelt et al. 1999) in the program Network v4.6.1 (http://www.fluxus-engineering.com). Networks allow for alternative potential evolutionary relationships to be shown as internal cycles. Median-joining networks incorporate median vectors which represent unsampled sequences or ancestral sequences that can allow for greater inference of genealogical relationships despite “missing” intermediary haplotypes.

### Population history

Variation in the HV II domain of the mtDNA control region was used to investigate historical demography after a pattern of expansion was suggested from the haplotype network analysis. As nuclear microsatellite data suggested weak population structure (see Sample collection and DNA extraction), we analyzed the mtDNA HV II data as the full dataset from all 15 swarming sites. First, we examined the distribution of the number of pairwise differences, the mismatch distribution, of samples to infer whether a recent and sudden expansion occurred (Rogers and Harpending 1992). The observed mismatch distribution was plotted against the expected values of a stable population (i.e., a population with constant population size). We also conducted three neutrality tests including Fu's F_S_ (Fu 1997) and Fu and Li' F* and D* statistics (Fu and Li 1993). All of these analyses were calculated in DnaSP (Librado and Rozas 2009). The F_S_ test has been shown to be a powerful test to detect population expansions (Ramos-Onsins and Rozas 2002) and is based on examination of the haplotype distribution where large negative values are expected under the scenario of expansion or alternatively from genetic hitchhiking resulting in a selective sweep. The statistical significance of this metric was evaluated by running 5000 coalescent simulations in DnaSP to create an expected distribution, and then comparing the observed value to these expected values. Comparing different neutrality tests can distinguish between the processes of a population expansion, genetic hitchhiking, or background selection. If Fu's F_S_ is significant but Fu and Li's F* and D*are not significant, then a population expansion is inferred over background selection where the former is observed as an excess of recent mutations and the latter as a deficiency of recent mutations (Fu 1997).

To infer the timing of the expansion, a Bayesian skyline plot was constructed using the coalescent model in the program BEAST v1.8.0 (Drummond et al. 2005). In trial runs, we initially tested three substitution models (HKY, GTR, and TN93) with 4 variants of site heterogeneity parameters for a total of 12 models within BEAST. We then used a Bayes factor assessment in TRACER (Rambaut and Drummond 2007) to find the best fit substitution model for the data. For the Bayesian skyline plot analysis, we subsequently used the TN93 + G model (Tamura and Nei 1993), with a lognormal relaxed clock run for 3.0 × 10^8^ steps, sampling every 1000 steps. Two independent chains were run, and the results were combined in LogCombiner as offered with the BEAST package. These parameters were found to be sufficient for convergence in trial runs as ESS parameters were >200 as viewed in TRACER. We used the range of divergence rates estimated for the HV II region in another temperate bat, *Nyctalus noctula* (6.5–25.2%; Petit et al. 1999), to estimate the rate in BEAST for our *M. lucifugus* sequences as no estimates exist for this species.

### Microsatellite genotyping

All samples were genotyped at 10 microsatellite loci previously described for this species (Table S1, Appendix; Burns et al. 2012). Briefly, loci were amplified in four multiplex reactions with optimized primer concentrations and polymerase chain reaction (PCR) annealing temperature. Reaction volumes were 10 *μ*L containing reagents as described above for the mitochondrial work with primer concentrations varying per locus in each multiplex (Table S1, Appendix). Each forward primer was labeled with one of four fluorescent dyes (NED, 6-FAM, VIC, PET^©^; Life Technologies, Carlsbad, CA, USA). Amplification conditions for PCR were as follows: an initial denaturing cycle of 95°C for 5 min; followed by 30 cycles of 95°C for 30 sec, annealing temperature for 1 min, 72°C for 1 min; with a final extension period of 64°C for 45 min. Cycling was carried out on Applied Biosystems (Carlsbad, CA, USA) 96 Well Veriti Thermal Cyclers, and amplified products were size-separated and visualized on an ABI 3500xL capillary electrophoresis system. Alleles were scored using GeneMarker (vs.1.95, SoftGenetics Inc., State College, PA) by comparison with GeneScan 600 LIZ® internal lane size standard (Life Technologies). For each individual, all loci electropherograms were visually inspected for verification of allele peak size calling; allele peaks were binned for scoring after examination of frequency distributions of raw allele calls. A negative and positive sample control (i.e., the same individual) was used on each 96-well plate run to ensure typing consistency among runs.

### Tests of assumptions and genetic structuring on nuclear DNA

Microsatellite loci were tested for departure from Hardy–Weinberg equilibrium (HWE) across each locus and within each swarming site using the Markov chain method in GENEPOP v4.1.3 (Raymond and Rousset 1995). Loci were checked for linkage disequilibrium in GENEPOP. Observed (H_O_) and expected heterozygosities (H_E_), number of alleles observed (N_A_) per population, and null allele frequency per locus were assessed using CERVUS v3.0.3 (Kalinowski et al. 2007); F_IS_ and allelic richness were calculated using FSTAT v2.9.3 (Goudet 1995). To complement adult nuclear analyses and assess temporal stability of genetic differentiation estimates, we calculated F_ST_ for two cohort sets of YOY for samples collected in 2009 (*n* = 69; 7 swarming sites) and 2010 (*n* = 102; 11 swarming sites).

To examine genetic differentiation among all swarming sites, *F* statistics were obtained by calculating overall and pairwise *F*_ST_ (FSTAT; Goudet 1995). A test for heterozygote deficiency was performed in GENEPOP. A Bayesian model-based clustering analysis was implemented using program STRUCTURE (v2.3.4) to infer the number of distinct genetic clusters within the nuclear dataset (Pritchard et al. 2000; Falush et al. 2003). Iterations were run without any *a priori* population information incorporated, using the admixture model with correlated allele frequencies among groups (Falush et al. 2003). Ten replicate runs were performed for *K* = 1–15 (the maximum number of swarming sites) with a burn-in of 500,000 steps and 2,000,000 recorded for the Markov chain Monte Carlo (MCMC) steps. We examined the ln probability, ln[P(X|*K*)], of the ten runs for each value of *K* (Pritchard et al. 2000) to evaluate the most probable number of genetic clusters (i.e., subpopulations) in the data.

Spatial analyses of genetic structure were conducted by performing an isolation-by-distance analysis (IBD) to test for correlation between geographic distance and genetic differentiation using a Mantel test implemented in the web-based IBDWS (v3.23; http://idbws.sdsu.edu/∼idbws/). Pairwise *F*_ST_ values were converted to (*F*_ST_ / (1 − *F*_ST_)) following Rousset (1997), and the log of the geographic distances (straight-line linear distances) was used where geographic coordinates were determined at each site using a global positioning system (GPS). Similar to the mitochondrial data, we conducted an AMOVA for the full nuclear dataset on all adults.

In addition, we examined population structure by examining relatedness within swarming sites implemented in the program STORM (Frasier 2008). This program calculates the pairwise relatedness coefficient of Li et al. (1993) with the weighting by locus scheme of Lynch and Ritland (1999) and Van de Casteele et al. (2001). A relatedness coefficient was calculated for all pairs within each swarming site, and the average was calculated within and across all swarming sites. To test whether the average relatedness at swarming sites differs from expectations of random grouping (i.e., from individuals from any swarming site), individual genotypes were shuffled 999 times between swarming sites keeping sample sizes the same to create a distribution of expected relatedness values from randomly associating individuals to estimate *P*-values.

Lastly, we tested for sex-biased dispersal by investigating F_ST_ and relatedness within each sex using the method described by Goudet et al. (2002). We considered females to have higher site fidelity fitting with the generalized pattern of male sex bias in mammals and previous work on temperate bats including specifically on *M. lucifugus* (Greenwood 1980; Kerth et al. 2002; Chen et al. 2008; Dixon 2011). Therefore, *F*_ST_ among sites and relatedness within sites are expected to be larger for females, the sex with the greater tendency to exhibit site fidelity. To test whether these metrics statistically differed between the sexes, we used the randomization approach implemented in FSTAT (10,000 permutations) where sex was randomly assigned to individuals within each subpopulation holding the number of each sex constant.

## Results

### mtDNA genetic variation

Ninety-five haplotypes were obtained from 356 adult individuals sampled across the fifteen swarming sites. Fifty-three polymorphic sites defined the haplotypes arising from 45 transitions, eight transversions, and two insertion/deletion events. Many haplotypes were found in only single individuals (56.8%). After correcting for sample size, we found that at the regional level, Quebec had the highest proportion of unique haplotypes (37.7%, *n* = 69) followed by New Brunswick (27.3%, *n* = 55) and Nova Scotia (17.7%, *n* = 226). Four haplotypes were found in high frequency (*n* = 30 or greater), with one found at 13 swarming sites (MYLU002, *n* = 77) and two found at 12 swarming sites (MYLU006, *n* = 35; MYLU007, *n* = 30). Both of these haplotypes were found in all three provinces. The remaining high-frequency haplotype (MYLU018, *n* = 48) was found at 10 swarming sites, 9 of which were in Nova Scotia and at one site in New Brunswick where only one individual with this haplotype was found. Haplotype diversity (*h*) was relatively high averaging 0.8523 ± 0.0981 (SD) and ranging from 0.8552 ± 0.0804, 0.8857 ± 0.1731, and 0.9073 ± 0.0285 for Nova Scotia, New Brunswick, and Quebec, respectively. Nucleotide diversity (*π*) was generally low and similar across all swarming sites averaging 0.0150 ± 0.0028 (SD) with a range of 0.0094 to 0.0184, although by province it was highest in Quebec followed by Nova Scotia and New Brunswick at 0.0155 ± 0.0029, 0.0154 ± 0.0025, 0.0132 ± 0.038, respectively.

In analyses of each sex, 57 haplotypes were found in 160 females, and 71 haplotypes were found in 196 males. Females exhibited a trend of higher variation in haplotype diversity among provinces whereas males exhibited more similar haplotype diversity among provinces (Table S2, Appendix) although these differences do not appear to differ greatly in magnitude; we did not test for significant differences. The pattern of variation in *π* among provinces was consistent for females, but for males it was highest in Nova Scotia followed by Quebec and New Brunswick.

### mtDNA population structure and demographic history

**Figure 3:**
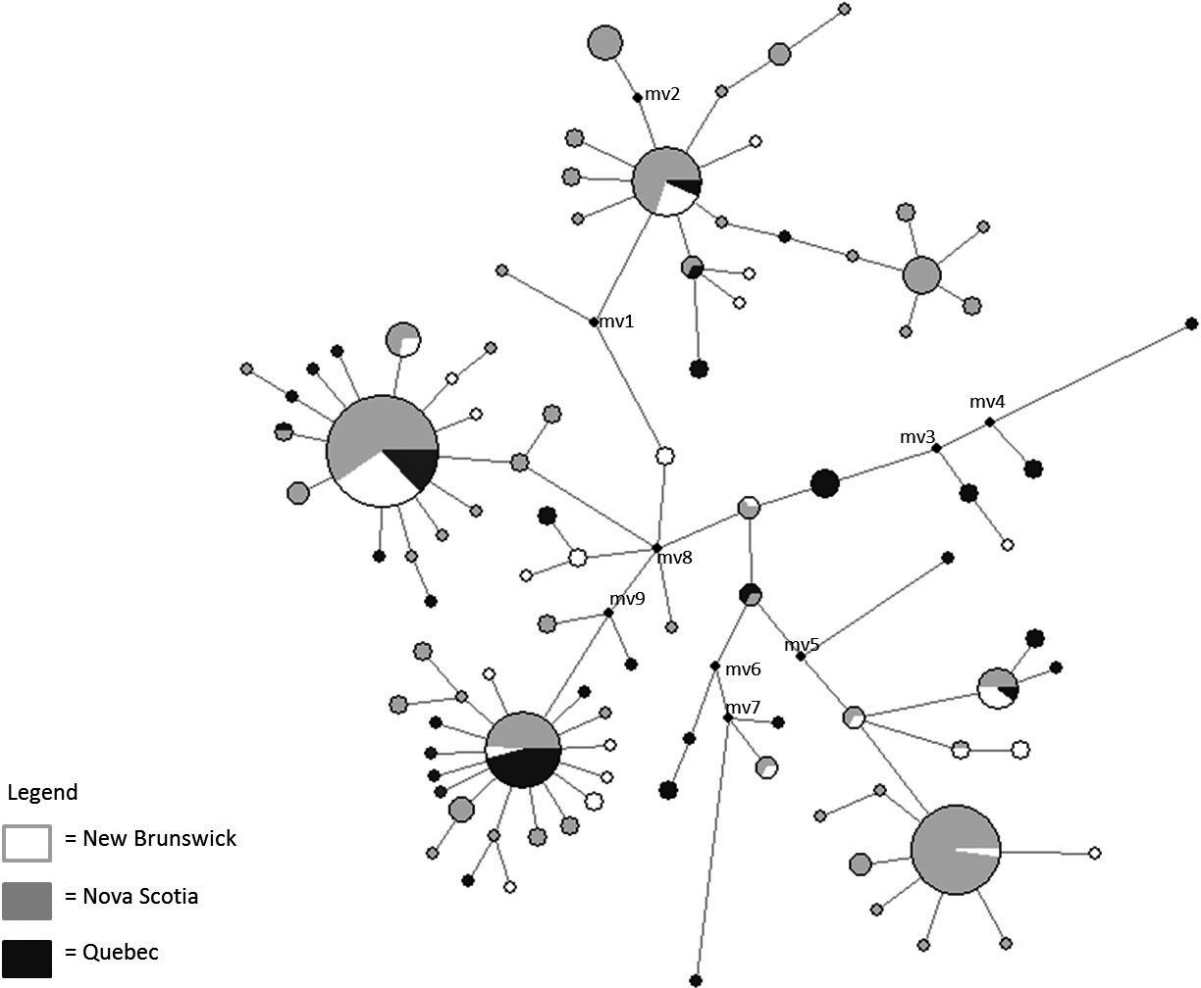
A median-joining network for *Myotis lucifugus* based on a 292-base-pair mitochondrial DNA segment of the control region coded by province. Circle size corresponds to haplotype frequency with inferred hypothetical haplotypes (mv) not sampled in the current study shown.

Structure inferred from mitochondrial data was an order of magnitude stronger than that from nuclear data (see below), but still indicative of low levels of population differentiation. Twenty-eight of the 105 pairwise comparisons (25.7%) of Φ_ST_ were significant, after correction for multiple tests, and the global Φ_ST_ estimate for all adults was 0.045 (*P* < 0.001). Analyzed separately, males and females had similar global Φ_ST_ estimates of 0.045 and 0.052 (both *P* < 0.001), respectively. Hierarchical analysis by AMOVA found that the majority of mitochondrial genetic differences were found within swarming sites (91.4%) and only 3.05% (*P* < 0.0001) of the variation found among provinces suggesting low regional structuring. The median-joining network (Fig. 3) demonstrated the lack of strong structuring by swarming site or by region (province) where many haplotypes were shared among sites and provinces with no distinct clustering of haplotypes by site. Low structuring within the network is suggestive of high historical gene flow. We defined an overall pattern of seven haplogroups radiating off of an unsampled intermediate haplotype in the center with several smaller haplogroups showing a starlike pattern of many single-nucleotide substitution haplotypes off of these larger central, high-frequency haplotypes; this pattern is indicative of a population expansion (Avise 2000).

**Figure 4:**
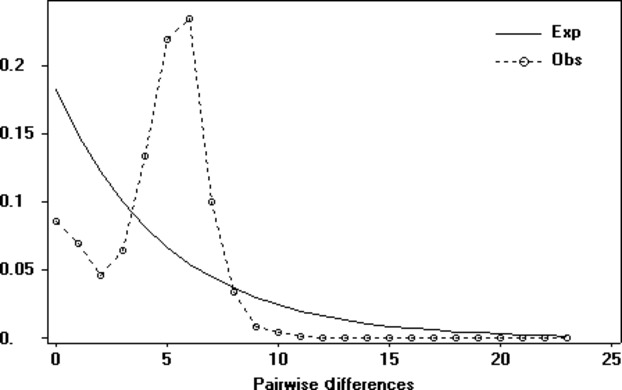
Mismatch distribution of *Myotis lucifugus* based on a 292-base-pair segment of the mitochondrial control region showing the observed frequency of pairwise differences among sequences (hatched line). The expected distribution (solid line) is for a population of constant size.

**Figure 5:**
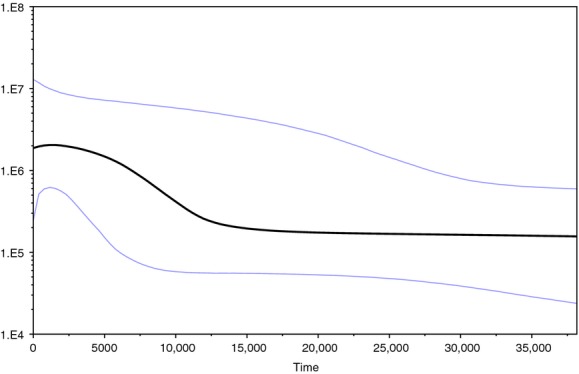
Bayesian skyline plot of the changes in effective population size backwards in time for *Myotis lucifugus* sampled from swarming sites in southeastern Canada. The *x*-axis represents time measured in years and the *y*-axis the population size (logarithmic) expressed as the product of the effective population size and the generation time in years (N_*e*_*τ*).

The pairwise comparison of all samples yielded a mismatch distribution of a unimodal peak that fits with a model of population expansion (Fig. 4). Fu's *F*_S_ statistic was statistically significant at *F*_S_ = −99.87 (*P* < 0.001), and (*P* = 0.11) and Fu and Li's F* and D* were not significant (F* = −2.052, *P* > 0.10; D* = −2.077, *P* > 0.10). The shape of the Bayesian skyline plot (BSP) suggests a similar population history to that of the mismatch distribution with an inferred population expansion (Fig. 5). The mean estimated divergence rate for the sequences was 15.7%/Myr with a mean likelihood of −1322.98. The BSP suggests *M. lucifugus* experienced a demographic expansion between 1250 and 12,500 before present, in the spatial region of our sampling.

### Nuclear DNA genetic variation

Of the 10 microsatellite loci genotyped, one locus (*Mluc*30) was removed from subsequent analysis because of significant deviations from Hardy–Weinberg equilibrium (HWE) across all swarming sites. Eight of remaining nine loci (without inclusion of comparisons of *Mluc*30) generally met the assumptions of HWE (Table S3) with Mluc5 showing deviations from HWE in six of the 15 sites and a null allele frequency estimation of 6.3%. Locus Mluc21 showed deviations from HWE in 13 of 15 sites and a null allele frequency estimate of 31.5%. We chose to retain this locus for analyses after an exploratory run with locus Mluc21 omitted produced similar results for pairwise *F*_ST_ estimates, *F*_IS_ estimates, and the test for overall heterozygote deficiency. Although null alleles can reduce genetic diversity resulting in increased *F*_ST_ estimates (Paetkau et al. 1997; Chapuis and Estoup 2007), our calculation of *F*_ST_ averaged the estimate overall all loci which should reduce this bias. The assumptions of linkage equilibrium were generally met with only 4 of the 40 comparisons deviating from linkage equilibrium, after Bonferroni correction. The genotyping error rate for the 9 loci, calculated from duplicate runs and analysis of 61 individuals (8% of the dataset), ranged from 0 to 3.3% per locus with a mean error rate of 1.6% although we did not perform a blind test of this. We retained 735 adults and 168 YOY for analyses that were successfully genotyped at ≥ seven of the nine loci. Mean observed heterozygosity was moderately high for adults (Table 2), generally similar across swarming sites (0.686 ± 0.018 SD), and was similar to levels found in YOY sampled in 2009 (0.716 ± 0.065 SD; Table S4, Appendix) and 2010 (0.730 ± 0.067 SD). Similar allelic-richness values were observed across all three provinces for adults (6.15–7.15).

**Table 2:** Genetic variation descriptors at 9 microsatellite loci and a 292-bp fragment of the mitochondrial DNA control region in adult *Myotis lucifugus* in southeastern Canada including the mean number of alleles per locus (A/locus), allelic richness (AR), observed heterozygosity (H_*O*_), expected heterozygosity (H_*E*_), within-site inbreeding coefficient (*F*_IS_), haplotype diversity (*h*), and nucleotide diversity (*π*)

Site	Nuclear microsatellite data					Mitochondrial control region		
	
	A/locus	AR	*H*_*O*_	*H*_*E*_	*F*_IS_	Number haplotypes	*h*	*π*
1	9.3	6.86	0.653	0.812	0.145	9	0.869	0.0173
2	11.2	6.83	0.670	0.804	0.105	10	0.788	0.0140
3	12.2	6.81	0.692	0.792	0.081	18	0.958	0.0184
4	13.4	7.02	0.710	0.822	0.080	10	0.850	0.0152
5	10.6	6.72	0.671	0.807	0.084	14	0.897	0.0171
6	11.4	7.05	0.687	0.817	0.093	15	0.897	0.0177
7	8.0	6.87	0.674	0.823	0.133	6	0.681	0.0117
8	12.2	6.67	0.682	0.800	0.099	15	0.908	0.0153
9	11.9	6.74	0.697	0.805	0.079	16	0.850	0.0117
10	10.1	6.74	0.709	0.803	0.077	17	0.940	0.0169
11	10.7	7.15	0.697	0.828	0.111	13	0.825	0.0134
12	6.6	6.15	0.667	0.779	0.106	3	0.600	0.0094
13	12.2	7.06	0.696	0.827	0.095	14	0.892	0.0123
14	9.6	6.76	0.714	0.824	0.056	16	0.940	0.0183
15	8.0	7.12	0.667	0.801	0.090	10	0.890	0.0158

### Nuclear DNA population structure

Metrics of population structure on nuclear markers indicate weak population differentiation. Of the 105 pairwise *F*_ST_ values from microsatellite data, only 1 was found to be significant (Table 3), and the global *F*_ST_ estimate was 0.001 ± 0.001 SE (*P* = 0.02). When analyzed separately, female and male adult bats also displayed a similar magnitude of global *F*_ST_ at 0.003 ± 0.002 SE (*P* = 0.003) and 0.001 ± 0.001 SE (*P* = 0.002), respectively. Despite the smaller sample sizes, the two cohort groups composed of YOY displayed a similar order of magnitude of global F_ST_ estimates to that found for the adult dataset (2009: *F*_ST_ = 0.004 ± 0.004 SE; 2010: *F*_ST_ = 0.002 ± 0.003 SE). These low estimates suggest high contemporary gene flow among swarming sites or shared recent ancestry for each sex and age class. Estimates of *F*_IS_ for all swarming sites were positive (Table 2) with a global estimate of *F*_IS_ = 0.135 ± 0.049 (SE), and the global test for heterozygote deficiency was significant (*P* < 0.005). This suggests nonrandom mating may occur within sites although the presence of null alleles at some loci could also explain these positive *F*_IS_ estimates.

**Table 3:** Pairwise *F*_ST_ estimates for 15 swarming sites for *Myotis lucifugus* based on nuclear microsatellite variation (above diagonal), and pairwise Φ_ST_ estimates based on mtDNA control region (below diagonal)

Site	1	2	3	4	5	6	7	8	9	10	11	12	13	14	15
1		0.000	0.000	−0.001	−0.001	−0.002	−0.005	−0.001	0.000	−0.001	−0.003	−0.004	0.001	−0.004	0.001
2	0.035		0.002	−0.001	−0.001	−0.002	−0.001	0.001	0.000	0.003	0.002	0.000	0.006	−0.005	0.008
3	0.049	**0.113**		0.003	0.002	−0.001	0.003	0.003	0.001	−0.003	0.003	0.000	0.010	−0.001	0.009
4	−0.009	−0.002	0.064		0.003	−0.002	−0.003	0.001	−0.001	−0.002	−0.002	−0.004	0.001	−0.002	0.001
5	−0.006	0.018	0.017	−0.021		0.001	0.001	0.002	0.004	0.007	0.009	0.001	0.009	0.001	0.010
6	0.011	0.011	0.007	0.006	−0.016		−0.006	0.003	−0.002	−0.003	0.000	−0.004	0.005	−0.005	0.004
7	0.148	**0.297**	0.124	0.156	0.121	**0.162**		0.005	−0.004	−0.003	0.000	−0.006	−0.003	0.000	−0.001
8	0.074	0.116	−0.010	0.062	0.014	0.007	0.094		**0.005**	0.000	0.001	0.000	0.003	−0.001	0.008
9	0.097	−0.015	**0.195**	0.066	**0.087**	**0.076**	**0.420**	**0.215**		−0.004	0.000	0.001	0.005	0.001	0.003
10	0.047	−0.057	0.189	0.020	0.055	0.051	0.432	**0.218**	−0.081		−0.003	−0.003	0.001	−0.012	0.004
11	0.006	0.010	0.036	0.001	−0.003	−0.003	**0.193**	0.051	0.063	0.024		−0.005	0.000	−0.004	0.000
12	**0.090**	−0.016	**0.138**	0.065	0.070	0.032	**0.372**	**0.151**	−0.002	−0.029	0.036		0.004	−0.007	0.001
13	0.063	0.085	**0.086**	0.077	0.060	0.062	**0.271**	**0.128**	**0.107**	0.064	0.022	**0.090**		0.009	0.004
14	0.068	**0.153**	0.094	**0.130**	0.106	**0.101**	**0.344**	**0.179**	**0.172**	0.145	0.060	0.151	0.007		0.008
15	0.062	−0.003	**0.134**	0.053	0.056	0.039	**0.379**	**0.164**	−0.004	−0.038	0.015	−0.009	0.036	0.083	

Swarming site codes are given in Table 1. Bold numbers indicate significant after Bonferroni corrections.

Low variance in the ln[P(X|*K*)] from the STRUCTURE analysis, across the replicates demonstrated convergence of the chains and indicated that *K* = 1 (mean ln[P(X|*K*)] = −24912.98 ± 0.175 (SD)), was the most likely number of genetic clusters represented in the data (Figure S1, Appendix). No evidence of correlation between geographic distance and genetic differentiation (isolation by distance) was found (*r* = 0.200, *P* = 0.877). Hierarchical analysis by AMOVA of microsatellite data indicated that the majority of genetic variation was found within swarming sites at the individual level (84.8% *P* < 0.001; Table 4) with low but significant variation found among swarming sites within provinces (15.0% *P* < 0.001) and very low variation found among provinces (0.15%, not statistically significant). Analyzed separately, adult males and adult females showed similar patterns to all adults together with only 0.11 and 0.08% of the variation found among provincial regions for males and females, respectively, supporting the lack of isolation-by-distance pattern.

**Table 4:** Hierarchical analysis of molecular variance (AMOVA) among mtDNA control sequences (Φ_ST)_ and 9 nuclear microsatellite loci (*F*_ST_) of *Myotis lucifugus* with regional (provinces) groupings. Percentage of the variation is for the three hierarchical levels

Source of variation	Φ_ST_ Sum of squares	Variance components	Variation (%)	F_ST_ Sum of squares	Variance components	Variation (%)
All adults						
Among provinces	25.455	0.075	3.05	11.5	0.005	0.15
Among swarming sites w/n provinces	64.746	0.136	5.53	3079.5	0.550	15.02
Within swarming sites	763.67	2.240	91.42	2283.5	3.107	84.83
Total	853.871	2.450		5374.5	3.662	
Females						
Among provinces	14.594	0.090	3.75	9.2	0.003	0.08
Among swarming sites w/n provinces	43.16	0.138	5.74	1174.8	0.597	16.24
Within swarming sites	314.284	2.167	90.51	855.5	3.077	83.67
Total	372.038	2.395		2039.5	3.678	
Males						
Among provinces	17.078	0.076	3.02	9.9	0.004	0.11
Among swarming sites w/n provinces	48.676	0.141	5.62	1891.2	0.520	14.26
Within swarming sites	413.941	2.287	91.36	1428.0	2.125	85.63
Total				3329.1	3.649	

Average pairwise relatedness among individuals within swarming sites was low with a mean *r* of −0.015 (range: −0.061 to 0.033 per site) within all sites. The mean expected within swarming site pairwise relatedness in 1000 permutations was −0.015, and therefore, the observed mean was not significantly different from simulated values of random groupings of bats (*P* = 0.532). Relatedness was similarly low for males and females when analyzed separately where no observed mean within swarming sites estimates were significantly different from random expectations after Bonferroni correction (Table S5; Appendix). In testing for sex-biased dispersal in FSTAT, we found stronger differentiation for females compared to males (*F*_ST_ females = 0.0033, *F*_ST_ males = 0.0004) and higher relatedness for females compared to males (females: 0.0059; males: 0.0008). However, these differences were not significantly different (*F*_ST_ *P* = 0.31; relatedness *P* = 0.30).

## Discussion

### Population structure

Fitting with our expectations of the high movement capability of the species, all lines of evidence from analysis of nuclear microsatellite data suggest weak population genetic structuring for *M. lucifugus* in southeastern Canada consistent with high gene flow. We found low global and pairwise *F*_ST_ among swarming sites with only one significant pairwise comparison suggesting that some weak structuring does occur. The significant comparison occurred between site 8 and site 9 where aside from this comparison, most other comparisons involving site 8 had higher estimates of F_ST_. This may reflect that this site was sampled more frequently than all other sites owing to other concurrent studies being conducted there which could have influenced the estimates of allele frequencies at the site. Higher sampling effort could result in the greater detection of rare alleles at this site relative to other sites such that it appears more differentiated. Regardless, the vast majority of pairwise comparisons were not significant suggesting high gene flow. Further support of high genetic connectivity comes from our STRUCTURE results which did not detect any genetic clusters within the data, and from the low estimates of pairwise relatedness among individuals that did not differ from expectations of free mixing of bats among swarming sites (i.e., random). Similar low estimates of relatedness at swarming sites were also found in three species of whiskered bats (genus *Myotis*) despite the presence of some pairs which may be full siblings (Bogdanowicz et al. 2012a). Genetic variation was higher within swarming sites compared to among sites which is consistent with the extra-colony hypothesis and may suggest some swarming site fidelity albeit with high genetic exchange among swarming sites. Lastly, an AMOVA did not detect large structuring at a regional spatial scale nor did we detect a significant isolation-by-distance (IBD) pattern.

In an analysis of summer captured individuals along riparian corridors, Lausen (2007) detected a significant IBD pattern in *M. lucifugus* which contrasts with a recent study of *M. lucifugus* maternity colonies where no significant IBD was detected (Dixon 2011); both studies occurred over a similar spatial scale (550–600 km) which were slightly smaller than ours (869 km). The extent to which an IBD pattern is displayed in bats tends to be stronger for more sedentary species compared to migratory species and depends on the spatial scale of sampling (Altringham 2011). However, it may also depend on landscape structure and context as availability and connectivity of habitat resources (e.g., foraging, roosting, commuting, or swarming sites) can influence movements, dispersal, and ultimately gene flow as shown in other mammals (Coulon et al. 2004; e.g., Broquet et al. 2006). The study by Lausen (2007) occurred in a prairie/agricultural landscape with sampling along river systems. This context may have restricted movements of individuals along these linear riparian features such that an IBD pattern was detected albeit within a larger framework of extensive gene flow similar to that characterized in ours and the Dixon (2011) study. Our study occurred primarily in the Atlantic Maritime Ecozone which is characterized by extensive forest cover (76%; McAlpine and Smith 2010) and also occurred during the autumn swarming and migration period where movements are expected to be greater. This contrasts the timing and landscape of Lausen's study.

The results from our mitochondrial DNA analyses also showed low levels of genetic structuring and further suggest a recent history of high gene flow in our sampled region. No structuring associated with geography at multiple spatial scales was detected from the hierarchical AMOVA. The median-joining network displayed little structuring of haplotypes by swarming site with many high-frequency haplotypes found at multiple sites and no clustering in any areas of the network by individual sites. In examining the network at the provincial level, there is still little evidence for strong structuring with 6 of the 7 haplogroups containing sequences found in ≥2 provinces with the exception of the singleton found in Nova Scotia. Site 7 stands out as having some of the highest Φ_ST_ values with other close by swarming sites in Nova Scotia and Quebec. This site was sampled on only two nights in 1 year, 2010, whereas most other sites were sampled more frequently (multiple nights in multiple years). If bats on a given night represent a small proportion of those swarming over the season and these bats are from the same summering colony/area that share common ancestry, this could explain these results.

Taken together, our results are consistent with weak genetic structuring as has been observed in other bat species known to swarm such as *M. nattereri* (Rivers et al. 2005), the three species of the *M. mystacinus* species complex (Bogdanowicz et al. 2012a), and *Plecotus auritus* (Furmankiewicz and Altringham 2007). Consistent with the extra-colony hypothesis, swarming appears to facilitate gene flow among segregated behavioral summer groups with recent work demonstrating greater genetic diversity and lower relatedness at swarming sites relative to summer maternity colonies (Veith et al. 2004; Furmankiewicz and Altringham 2007; Kerth et al. 2008). This suggests bats from multiple colonies meet at swarming sites but may not necessarily mate there. However, using simulations, Rivers et al. (2005) found that in the swarming *M. nattereri*, the levels of observed population structure were most consistent with a model with effective mating occurring at swarming/hibernation sites rather than within summer colonies. In conjunction with behavioral studies documenting mating activities at swarming sites (Barclay and Thomas 1979; McGuire et al. 2009; Furmankiewicz et al. 2013), this supports the contention that swarming sites are “hot spots” for gene flow (Kerth et al. 2003).

Bat species that make extensive migratory movements or have large dispersal capacities can be characterized by near panmictic genetic structures such as *Tadarida brasiliensis* (McCracken et al. 1994; Russell et al. 2005a), *N. noctula* (Petit and Mayer 1999), and *Pipistrellus pipistrellus* and *P. pygmaeus* (Bryja et al. 2009). It is important to note that regional differences in the magnitude of population genetic structure can also be found in some migratory species owing to different landscapes and resultant migratory behavior (Bryja et al. 2009; Sztencel-Jablonka and Bogdanowicz 2012). In *N. noctula* and another long-distance migratory Pipistrelle species (*P. nathusii*), mating takes place during migration (Petit and Mayer 2000; Petit et al. 2001; Hutterer et al. 2005) which may largely explain many of the low genetic structures observed in migratory species over great distances. We suggest that for *M. lucifugus* in our study area, the evidence of weak genetic structuring is likely due to a combination of swarming behavior, which facilitates dispersal among segregated winter and summer groups, and the high movement capability of *M. lucifugus* due to migration during this period. Although mating may occur outside of the swarming period for *M. lucifugus* (Fenton 1969; Thomas et al. 1979), and recent work in the swarming *M. daubentonii* has shown mating to occur at summer sites (Senior et al. 2005; Angell et al. 2013), further work would be required to assess the importance of mating activities occurring away from swarming sites to the overall mating strategies and contributions to gene flow in *M. lucifugus*. In a study of *M. nattereri*, Rivers et al. (2005) found that swarming sites show genetic distinctiveness over a smaller geographic range than our study area and suggested that bats from a given summer colony show high swarming site fidelity. This differs from *M. lucifugus* that appear to show less swarming site fidelity being more transient in their swarming movements (Humphrey and Cope 1976; Norquay et al. 2013) compared to *M. nattereri*.

*Myotis lucifugus* is thought to display the typical mammalian pattern of male natal dispersal (Greenwood 1980) with males generally not returning to their natal maternity colonies to associate closely with females in subsequent years (Davis and Hitchcock 1965; Fenton 1969; Frick et al. 2010b). As swarming in bats may function as a form of temporary dispersal facilitating gene flow among individuals segregated during the summer, characterizing the extent of sex-biased dispersal in determining biases in sex-directed gene flow may be informative at swarming sites when most mating is thought to occur. We did not find evidence to suggest strong asymmetries in gene flow between the sexes on biparentally inherited nuclear markers. However, it is important to keep in mind that the method we used detects recent differences in dispersal (Prugnolle and de Meeus 2002). Further, it makes many assumptions such as sampling after dispersal has occurred, which is problematic in species with overlapping generations, and works best under scenarios of strong sex biases in dispersal (Goudet et al. 2002) which may not be the case for *M. lucifugus*.

Movement data from recapture studies during the swarming period are scarce but there are occurrences of large movements by both sexes. Work from Manitoba and Ontario (Canada) for *M. lucifugus* showed two females and three males were captured visiting multiple swarming sites (Norquay et al. 2013). This and other studies have shown both sexes swarming and hibernating at different sites within and among years (Davis and Hitchcock 1965; Fenton 1969; Humphrey and Cope 1976). Autumn swarming appears to be a complex time for bats with individuals migrating to overwintering sites, increasing fat stores for hibernation, and mating. Thus, individuals may have multiple motivations impacting their decisions on their activities during this time stemming from differences in energy allocation (Kunz et al. 1998). For males, movements may primarily reflect mating choices in trying to maximize mating opportunities during swarming but may also represent movements made in selecting an optimal hibernation site. Females may more strongly select the later scenario over securing many mating opportunities compared to males. Regardless, if regular movements by both sexes ultimately contribute to gene flow, then this occurs among sites by both sexes during this temporary dispersal period and our data support this assertion. Inference of sex-biased dispersal can also come from comparisons of structure on markers with different modes of inheritance (i.e., mtDNA) which tend to reflect more long-term patterns of gene flow. We found stronger structuring on mtDNA compared to nuclear DNA which may suggest there is a male bias in gene flow long term as stronger differentiation on mtDNA is expected when females are more philopatric (Prugnolle and de Meeus 2002). However, regular movements by both sexes during swarming may reduce the magnitude of the bias as detected in the short term. Future work quantifying sex-specific demographic parameters to estimate the magnitude of the male-biased dispersal could be undertaken to better understand these dynamics such as done for *N. noctula* (Petit et al. 2001).

### Population history

Our data suggest that *M. lucifugus* in our study area experienced a population expansion since the last glaciation. This interpretation is supported by several lines of evidence including the starlike topology of the median-joining network, neutrality tests, mismatch distribution, and Bayesian skyline plot analysis. A significant neutrality test can suggest multiple scenarios including a population expansion, genetic “hitchhiking” by an advantageous mutation, or background selection. However, some of these various explanations can potentially be differentiated from each other by comparing different neutrality tests. For example, Fu and Li's F* and D* statistics (Fu and Li 1993) are more strongly affected by background selection relative to Fu's F_S_ statistic (Fu 1997) which is more strongly affected by population expansion or selective sweeps. In comparing the two, a significant *F*_S_ and nonsignificant F* and D* indicate an excess of singleton haplotypes which favors a scenario of a population expansion or a selective sweep rather than background selection which our data show. We cannot rule out the possibility of a past selective sweep that replaced all mtDNA haplotypes which was then subsequently followed by an accumulation of neutral variants from that haplotype (Maruyama and Birky 1991). However, the concordance of this expansion scenario with other supporting analyses strongly supports a population expansion as does additional information from the molecular diversity indices of the mtDNA data.

Haplotype diversity (*h)* in the HV II region was relatively high, and nucleotide diversity (*π*) was low, a pattern which is consistent with a population expansion. A similar pattern of exceptionally high *h* and low *π* was described in the tropical Brazilian free-tailed bat (*T. brasiliensis)* which is thought to have undergone an expansion within the past 3000 years (Russell et al. 2005a). Our levels of *h* and *π* are more similar to those found in the temperate common noctule bat (*N. noctula*) where the inferred expansion followed the Younger Dryas period (12,900–11,500 BP; Petit et al. 1999). From the BSP with an estimated divergence rate of 15.7% / Myr, we estimate a population expansion occurring from approximately 12,500 to 1,250 BP which broadly correlates to recolonization of forests in the region that occurred following Pleistocene glaciation in North America. It is now thought that during the last glacial maximum (LGM; approximately 18 ka), the ice sheet in southeastern Canada extended close to the present day off-shore continental shelf. Ice-free areas extended south of the region along the coast in the United States with glacial refugia on present day George's Bank just south of Nova Scotia (Shaw et al. 2006). Although other off-shore refugia have been proposed and debated under alternate models of glacial reconstruction (Pielou 1991; Davis and Browne 1996), their occurrences may have been after the LGM (Shaw et al. 2006) or were short in duration following changing sea levels (Holland 1981; Shaw et al. 2002) such that they may not have supported extensive forest ecosystems to act as suitable refugia for bats. Following glacial retreat, forest recolonization is thought to have occurred from the south by 13 ka (summarized in Miller 2010) and the estimated population expansion for *M. lucifugus* follows this shortly thereafter. The high vagility of bats and the behavioral flexibility in roosting exhibited by *M. lucifugus* (Fenton and Barclay 1980) may mean that they could have closely tracked forest recolonization including use of early open-stand forests through to their replacement by closed-stand forests. The presence of caves containing fossil Quarternary mammals in an area just north of our study area (Gaspé, Quebec; Harington 2011) suggests that some underground sites have a long history of existence in the region that could facilitate the hibernation requirements of bats as they recolonized forested areas.

### Genetic connectivity and conservation implications

Our findings suggest a high degree of genetic connectivity in *M. lucifugus* with gene flow occurring from dispersal by both males and females, although it may be male-biased. Along with mutation and selection, gene flow is only one of the major forces that shape the genetic structure of species and the role of genetic drift must also be considered (Hartl and Clark 1997). Gene flow can counteract the effects of genetic drift by opposing the divergences that strong genetic drift reinforces. However, the effects of drift depend on effective population size (N_e_) where large N_e_ reduces the role of genetic drift. Key factors that influence N_e_ include population size and patterns of reproductive success (Allendorf and Luikart 2007). Bats can exhibit social structures and mating systems that can result in nonrandom mating leading to variation among individuals in reproductive success and potentially N_e_ despite large population abundances (Storz 1999). Although N_e_ has not been quantified for *M. lucifugus*, we expect it to be quite large (at least prior to WNS) based on large historical hibernating population estimates (Trombulak et al. 2001; Frick et al. 2010a; Turner et al. 2011) and linkages between many of the sites from banding work (Davis and Hitchcock 1965; Fenton 1970; Humphrey and Cope 1976) similar to that shown in *T. brasiliensis* (Russell et al. 2005a). With a large N_e_, low levels of genetic structure are expected even with low genetic exchange and future work that characterizes this parameter would provide valuable insight into the genetic structuring of *M. lucifugus*, particularly in light of the large population declines from WNS.

The implications of high genetic connectivity in managing populations under the epizootic of WNS remain complicated and largely unknown. High genetic connectivity may imply dispersal, whether permanent or temporary, over extensive spatial scales. As recent work has shown bat-to-bat transmission in a laboratory setting (Lorch et al. 2011), it is possible that these movements, if the bats are infectious at the time, could be contributing to the rapid spread of the disease. Although this has not been tested, bats have many opportunities for direct contact with other bats during swarming due to mating and potentially information-sharing activities occurring within and around underground sites. These activities could facilitate transmission of spores among bats if these sites act as environmental reservoirs of *P. destructans* (Lindner et al. 2011). Taken together with evidence of large movements from recapture data from swarming and among hibernation sites (Humphrey and Cope 1976; Norquay et al. 2013), this high level of connectivity may partially explain the rapid spread of the disease. Recent work has shown correspondence among genetic structure and the spread of WNS in Pennsylvania in *M. lucifugus* which may reflect movement patterns of bats (Miller-Butterworth et al. 2014). In our study area, WNS was detected first in Quebec followed by detection in the neighboring provinces of New Brunswick and Nova Scotia a year later. The total spread of the disease in North America from discovery in 2006 is in excess of 2000 km. With no effective means to control the spread of the disease thus far, further work assessing connectivity, both genetic and demographic, within other areas of the species range may be able to provide information in the short term on transmission dynamics and spread. However, this knowledge may also be used to inform pertinent demographic questions on survival, immigration, and emigration rates as they relate to future population persistence and connectivity of local populations (Lowe and Allendorf 2010).

In summary, our findings suggest high gene flow and therefore high genetic connectivity among swarming sites of *M. lucifugus* in southeastern Canada. We found no evidence to suggest a strong signature of structure but rather found evidence of a demographic expansion following deglaciation of the region. Although our study suggests dispersal over a large spatial scale in the recent past, predicting how the dynamics of dispersal will contribute to the trajectory of population persistence in the future is not a straightforward process. Since the emergence of WNS, many local hibernating populations have been dramatically reduced in the eastern portion of the range (Frick et al. 2010a; Ingersoll et al. 2013), including our study area (L. E. Burns & H. G. Broders, unpubl. data). Future work should incorporate other approaches to characterize dispersal and other demographic parameters in addition to genetic data to allow predictions to be made on population viability in light of WNS. Although it was not an initial goal of our study, our data will provide a valuable baseline for future comparative studies of genetic structure and connectivity before and after a large mortality event. An understanding of the patterns of connectivity prior to such an event may enable such information to be incorporated into management plans for other regional populations prior to the arrival of WNS in those regions and in our own region in a post-WNS setting.

## Data Accessibility

Data are available from the Dryad Digital Repository: http://doi.org/10.5061/dryad.hs37s. Data include the microsatellite data as a GENEPOP input file, STRUCTURE input file and parameters, and DNA sequence data as an ARLEQUIN input file.

## Supporting Information

**Table S1.** Multiplex PCR conditions, loci specific fluorescent dye, observed number of alleles and allele size ranges (base pairs) for microsatellite loci used in genotyping *Myotis lucifugus* bats from south-eastern Canada. **Table S2.** Genetic variation in a 292-bp fragment of the mitochondrial DNA control region in adult male and female *M. lucifugus* in south-eastern Canada as haplotype diversity (*h*) and nucleotide diversity (π). **Table S3.** *P* values for deviation from HWE for each locus and swarming site sampled in genotyping *Myotis lucifugus* bats from south-eastern Canada. **Table S4.** Genetic variation descriptors at 9 microsatellite loci in young-of-the-year *M. lucifugus* bats in south-eastern Canada. **Table S5.** Average pairwise relatedness coefficients for individual *M. lucifugus* from swarming sites and the average across all swarming sites. **Figure S1.** Mean posterior probability of the data (Ln*P*(*D*) against the number of *K* genetic clusters within the data for 735 *M. lucifugus* sampled at 15 swarming sites in south-eastern Canada.
