# Supplementary material for: Genetic connectivity among swarming sites in the wide ranging and recently declining little brown bat (Myotis lucifugus)
Source: Ecol Evol. 2014 Oct 12;4(21):4130–49. doi: 10.1002/ece3.1266 (PMC4242565; doi:10.1002/ece3.1266)
Supplement: Supplementary file 1 [file ece30004-4130-SD1.docx]

**Appendix: Supplemental Information**

Table S1. Multiplex PCR conditions, loci specific fluorescent dye, observed number of alleles and allele size ranges (base pairs) for microsatellite loci used in genotyping *Myotis lucifugus* bats from south-eastern Canada.

| Locus | Fluorescent | Multiplex | Annealing | Primer concentration | Number | Allele sizes |
| --- | --- | --- | --- | --- | --- | --- |
|  | dye | Reaction | temperature (°C) | [µM] | of alleles | (bp) |
| *Mluc*1 | 6-FAM | 1 | 60 | 0.08 | 10 | 115-151 |
| *Mluc4* | VIC | 2 | 60 | 0.10 | 8 | 141-169 |
| *Mluc5* | 6-FAM | 2 | 60 | 0.10 | 10 | 132-172 |
| *Mluc7* | NED | 3 | 60 | 0.10 | 31 | 140-260 |
| *Mluc8* | PET ® | 2 | 60 | 0.20 | 28 | 145-285 |
| *Mluc11* | 6-FAM | 4 | 55 | 0.10 | 10 | 220-256 |
| *Mluc21* | 6-FAM | 1 | 60 | 0.20 | 5 | 303-319 |
| *Mluc25* | PET ® | 4 | 55 | 0.15 | 27 | 298-402 |
| *Mluc30* | 6-FAM | 3 | 60 | 0.15 | 59 | 266-402 |
| *Mluc34* | PET ® | 3 | 60 | 0.20 | 15 | 328-384 |

Table S2. Genetic variation in a 292-bp fragment of the mitochondrial DNA control region in adult male and female *M. lucifugus* in south-eastern Canada as haplotype diversity (*h*) and nucleotide diversity (π ).

|  | Males (*n* =196 ) | |  | |  | Females (*n* =160 ) | | |  |
| --- | --- | --- | --- | --- | --- | --- | --- | --- | --- |
| Site | Number of haplotypes | *h* | | π |  | | Number of haplotypes | *h* | π |
| 1 | 9 | 0.876 | | 0.0179 |  | | 3 | 1.000 | 0.0160 |
| 2 | 7 | 0.873 | | 0.0162 |  | | 5 | 0.709 | 0.0115 |
| 3 | 10 | 0.924 | | 0.0194 |  | | 12 | 0.967 | 0.0167 |
| 4 | 5 | 0.857 | | 0.0166 |  | | 9 | 0.876 | 0.0154 |
| 5 | 7 | 0.879 | | 0.0159 |  | | 11 | 0.933 | 0.0182 |
| 6 | 10 | 0.900 | | 0.0186 |  | | 8 | 0.901 | 0.0174 |
| 7 | 5 | 0.722 | | 0.0130 |  | | 3 | 0.700 | 0.0110 |
| 8 | 9 | 0.908 | | 0.0155 |  | | 11 | 0.933 | 0.0153 |
| 9 | 8 | 0.733 | | 0.0087 |  | | 10 | 0.934 | 0.0139 |
| 10 | 14 | 0.938 | | 0.0160 |  | | 6 | 1.000 | 0.0192 |
| 11 | 9 | 0.923 | | 0.0148 |  | | 7 | 0.724 | 0.0113 |
| 12 | 2 | 0.667 | | 0.0115 |  | | 2 | 0.667 | 0.0092 |
| 13 | 9 | 0.905 | | 0.0135 |  | | 8 | 0.859 | 0.0105 |
| 14 | 11 | 0.934 | | 0.0184 |  | | 8 | 0.956 | 0.0155 |
| 15 | 5 | 0.786 | | 0.0133 |  | | 6 | 1.000 | 0.0206 |

Table S3. P values for deviation from HWE for each locus and swarming site sampled in genotyping *Myotis lucifugus* bats from south-eastern Canada.

|  | Swarming site | | |  |  |  |  |  |  |  |  |  |  |  |  | Null |
| --- | --- | --- | --- | --- | --- | --- | --- | --- | --- | --- | --- | --- | --- | --- | --- | --- |
| Locus | 1 | 2 | 3 | 4 | 5 | 6 | 7 | 8 | 9 | 10 | 11 | 12 | 13 | 14 | 15 | allele frequency |
| Mluc1 | 0.569 | 0.029 | 0.478 | 0.295 | 0.337 | 0.214 | 0.250 | 0.434 | 0.893 | 0.790 | 0.760 | 0.012 | 0.471 | 0.739 | 0.415 | 0.011 |
| Mluc4 | 0.022 | 0.156 | 0.892 | 0.067 | 0.867 | 0.588 | 0.785 | 0.965 | 0.604 | 0.290 | 0.522 | 0.886 | 0.768 | 0.007 | 0.233 | 0.032 |
| Mluc5 | 0.605 | 0.577 | 0.145 | 0.014 | 0.029 | 0.217 | 0.000 | 0.221 | 0.011 | 0.740 | 0.079 | 0.443 | 0.000 | 0.046 | 0.361 | 0.063 |
| Mluc7 | 0.754 | 0.231 | 0.310 | 0.723 | 0.180 | 0.071 | 0.854 | 0.525 | 0.644 | 0.761 | 0.000 | 0.867 | 0.361 | 0.170 | 1.000 | 0.006 |
| Mluc8 | 0.972 | 0.426 | 0.900 | 0.069 | 0.053 | 0.157 | 0.485 | 0.138 | 0.116 | 0.122 | 0.500 | 0.012 | 0.544 | 0.595 | 0.010 | 0.024 |
| Mluc11 | 0.272 | 0.262 | 0.184 | 0.423 | 0.879 | 0.002 | 0.499 | 0.038 | 0.103 | 0.618 | 0.873 | 0.477 | 0.006 | 0.762 | 0.583 | 0.071 |
| Mluc21 | 0.000 | 0.000 | 0.000 | 0.000 | 0.001 | 0.002 | 0.002 | 0.000 | 0.000 | 0.001 | 0.000 | 0.090 | 0.000 | 0.103 | 0.015 | 0.315 |
| Mluc25 | 0.364 | 0.659 | 0.607 | 0.431 | 0.270 | 0.804 | 0.115 | 0.082 | 0.923 | 0.718 | 0.416 | 0.986 | 0.809 | 0.244 | 0.590 | 0.002 |
| Mluc34 | 0.124 | 0.646 | 0.009 | 0.853 | 0.789 | 0.218 | 0.277 | 0.081 | 0.916 | 0.463 | 0.089 | 0.981 | 0.279 | 0.953 | 0.914 | 0.014 |

Table S4. Genetic variation descriptors at 9 microsatellite loci in young-of-the-year *M. lucifugus* bats in south-eastern Canada. Measure include the mean number of alleles per locus (A/locus), observed heterozygosity (H*_O_* ), expected heterozygosity (H*_E_* ) and within site inbreeding coefficient (F_IS_).

|  | 2009 Juveniles (*n*= 67) | | |  | 2010 juveniles (*n* =101) | | | |
| --- | --- | --- | --- | --- | --- | --- | --- | --- |
| Site | A/locus | H*_O_* | H*_E_* | F_IS_ | A/locus | H*_O_* | H*_E_* | F_IS_ |
| 1 | 4.67 | 0.627 | 0.777 | 0.213 | 6.11 | 0.714 | 0.812 | 0.129 |
| 2 | 3.44 | 0.778 | 0.807 | 0.053 | 7.78 | 0.772 | 0.755 | -0.023 |
| 3 | - | - | - | - | 3.00 | 0.833 | 0.796 | -0.083 |
| 4 | 9.22 | 0.722 | 0.787 | 0.084 | 6.67 | 0.750 | 0.818 | 0.089 |
| 5 | 7 | 0.775 | 0.728 | 0.155 | 6.56 | 0.812 | 0.853 | 0.055 |
| 6 | 4.33 | 0.630 | 0.832 | 0.074 | 7.00 | 0.749 | 0.797 | 0.067 |
| 7 | - | - | - | - | 6.11 | 0.658 | 0.781 | 0.167 |
| 8 | 8.22 | 0.713 | 0.807 | 0.120 | 8.78 | 0.765 | 0.800 | 0.045 |
| 9 | - | - | - | - | 3.67 | 0.685 | 0.811 | 0.196 |
| 10 | - | - | - | - | 3.44 | 0.611 | 0.759 | 0.25 |
| 11 | - | - | - | - | 8.11 | 0.686 | 0.787 | 0.134 |
| 14 | 3.67 | 0.767 | 0.741 | 0.036 | - | - | - | - |

Table S5. Average pairwise relatedness coefficients for individual *M. lucifugus* from swarming sites and the average across all swarming sites. No coefficients were found to be significantly different from random groupings of bats across all swarming sites after Bonferroni correction.

| Site | All adults | Females | Males |
| --- | --- | --- | --- |
| 1 | -0.033 | -0.066 | -0.027 |
| 2 | -0.014 | 0.006 | -0.033 |
| 3 | -0.001 | 0.060 | -0.029 |
| 4 | -0.031 | -0.038 | -0.024 |
| 5 | -0.030 | -0.125 | -0.003 |
| 6 | -0.006 | 0.004 | -0.021 |
| 7 | -0.061 | 0.005 | -0.057 |
| 8 | 0.020 | 0.039 | 0.009 |
| 9 | 0.000 | 0.010 | -0.011 |
| 10 | 0.001 | 0.026 | -0.005 |
| 11 | -0.051 | -0.050 | -0.052 |
| 12 | 0.033 | -0.029 | 0.028 |
| 13 | -0.030 | -0.076 | 0.002 |
| 14 | -0.010 | -0.067 | 0.017 |
| 15 | -0.018 | -0.042 | -0.039 |
| *Average* | -0.015 | -0.023 | -0.016 |


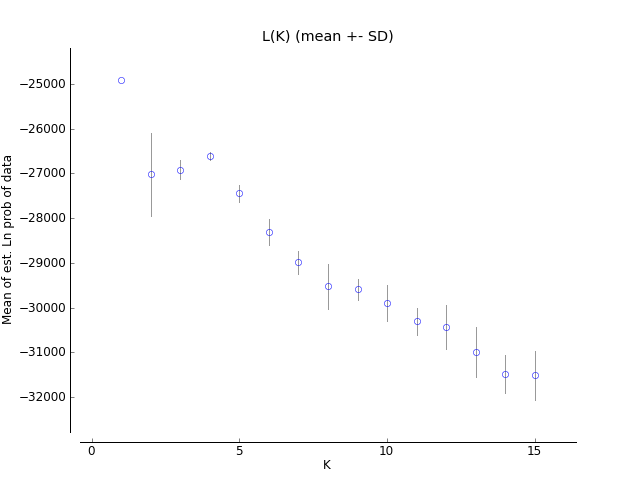


Figure S1. Mean posterior probability of the data (Ln*P*(*D*) against the number of *K* genetic clusters within the data for 735 *M. lucifugus* sampled at 15 swarming sites in south-eastern Canada.
